# Quantitative risk estimation of CNG station by using fuzzy bayesian networks and consequence modeling

**DOI:** 10.1038/s41598-024-54842-y

**Published:** 2024-02-21

**Authors:** Behzad Abbasi Kharajou, Hassan Ahmadi, Masoud Rafiei, Saber Moradi Hanifi

**Affiliations:** 1 Department of Urban Planning, University of Guilan, Rasht, Iran; 2 Department of Urban Planning, Faculty of Architecture and Art, University of Guilan, Rasht, Iran; 3 Department of Occupational Health, Faculty of Health, Ahvaz Jundishapur University of Medical Sciences, Ahvaz, Iran; 4 Department of Occupational Health Engineering, School of Public Health, Iran University of Medical Sciences, Tehran, Iran

**Keywords:** QRA, FFTA, Bow-tie, Bayesian network, CNG station, Health occupations, Chemical engineering

## Abstract

As one of the potential explosions and inflammation, compressed natural gas (CNG) stations in urban areas cause irreparable losses and casualties. Estimating risk assessment in gas stress based on coherent uses can reduce accidents in urban areas. The purpose of the present study was to estimate a small risk estimation at one of the CNG multipurpose stations, LPG, using combined models of the Fuzzy Bayesian Network, Bow-tie Diagram, and consequence modeling. This study was conducted based on the basic and 25 intermediate events. This study formed a seven-person safety team to identify the primary events and build the Bow-tie diagram. Then, because of the lack of a proper database, fuzzy theory was used to determine the probability of significant events. Bayesian networks were drawn based on the Bow-tie model using GeNLe software. Finally, the main events of the two Bow-tie, Bayesian network modeling, and risk estimation were performed with the help of PHAST/SAFETI (V8.22). The geographical information system software was used to zone the explosion effects. The Risk assessment result showed that the social risks and the Bayesian network model are more than Bow-tie, and the Bow-tie diagram is unacceptable. Therefore, using incompatible land uses in the vicinity of the CNG stations gives rise to the effects of accident scenarios in particular residential and administrative land uses, which decision-makers and city managers should consider. Based on the findings of this study, the obtained results can be utilized to implement effective control measures. These measures encompass devising a response plan tailored to address specific emergency conditions and conducting comprehensive training programs for the individuals and residents residing within the study area.

## Introduction

### Background

The positioning of some land uses within urban areas, disregard for the expansion of cities and improper locating of different land uses for the future development of urban areas are the underlying reasons that have contributed to explosions and many other hazardous incidents within urban areas^1,2^. Compressed natural gas (CNG) is the natural gas held in the compressive state within vessels. This type of gas is used in urban areas in CNG stations and service land uses. Natural gas is composed primarily of methane, and in the lower proportion of ethane propane, of different types of butane and other heavy alkanes. The flammability range of natural gas and air at atmospheric pressure is about 5–15% volume. The working pressure of a CNG station is 200–250 bars^3^. Numerous studies have been conducted despite the risk of explosion at gas stations. Grecea and colleagues conducted a study to provide helpful information to take optimal measures to minimize the risk of explosions at gas stations. They simulated the effects of each fuel based on the worst scenario using ANSYS software—Fluid Dynamics. The results showed that using replacement fuels could increase the risk of explosion compared to resonant fuels and need safety measures^4^. CNG stations represent one of the service land uses in cities, which are located in the vicinity of incompatible land uses while cities are developed and expanded around the neighborhood. Improper urban management, the invasion of some land uses to urban areas, Inflammation and explosion are factors contributing to the creation of Irreparable events. The effects of accident scenarios are defined as an incident that initially spreads to adjacent equipment and causes a series of secondary events^5^. In general, they are distinguished by the nature of hazards, which include natural and human hazards, followed by technological and organizational risks. These effects combine these two types of hazards^6^.

### Risk assessment

Specific techniques have been used to evaluate risk and several methods have been provided and developed by researchers for different conditions. Therefore, choosing a proper method is different based on the studied industries and goals of studies^7^. Several complementary risk evaluation methods in industrial processes are necessary, because it presents a clear picture of the work process and accident scenarios^8^. One of these uses is quantitative evaluation of risk two parameters play the main role in risk analysis. The first parameter is the probability of the occurrences and the second one is the severity of the consequence. The probability of the occurrence, probability of event occurrence or defect leads to an event over a period of time. The intensity of consequences of an event means the harmful effects caused by accident^9^. Scenario can have several consequences that these consequences are determined and simulated through the presented models for "release and diffusion of substances in the environment" and "fire" and "explosion" models. The effects caused by fire are determined as the distribution of radiation intensity and the effects caused by explosion are determined as the distribution of explosion wave and the effects of these consequences on the human population are evaluated^9^.

### Fuzzy Bow-tie diagram

The Bow-tie analysis shows risk and states a range of possible causes and consequences. This model analyzes an affordable approach to evaluate process risk. This approach is a qualitative Evaluation Technique that is ideal for the initial analysis of an existing process or application in the middle process of the design of the process^10^. The benefits of this technique include simplicity, visual expression, and focus on existing controls for the prevention and effectiveness of these controls^10,11^. The Bow-tie method is a great model for analyzing refinery process accidents. This model can be used for the release of natural gas leakage. Whether qualitative or quantitative, this method is a helpful tool for determining the causes of events and identifying them^12^. Different studies were carried out to analyze the risk of natural gas by the Bow-tie model. Muniz et al. presented a risk management method for natural gas pipelines using the Bow-tie diagrams. The results showed that the Bow-tie model is a valuable risk management method for natural gas pipelines and can be one of them. Used to identify deficiencies and propose recommended measures to improve safety measures^13^. Sharriar and colleagues used the Fuzzy and the Bow-tie Diagrams to analyze the oil pipeline and gas risk. This study showed that this method can provide preventive or corrective measures and conscious decisions in the risk management process for oil and gas companies^14^. In Bow-tie method, databases, such as Odisha Renewable Energy Development Agency (OREDA) O. R. D. Handbook, OREDA Participants, Orissa Renewable Energy Development Agency, Bhubaneswar, India, 2002, Center for Chemical Process Safety^15^ and Mannan^16^ can be used to determine the probability of basic events. However, the use of databases is limited due to the lack of failure rate for all root events and also the low reliability of related data. In some sources, such as the NOG-070 report, it is mentioned that if the number of components prone to defects is less than 30 components, the estimated defect rate will not be reliable to assess the safety of the system^9^. Using conventional methods to quantify the probability of basic events in incidents involving CNG Tanks requires valid databases that are not usually in fuel stations. A fuzzy approach is used to solve this problem. Fuzzy logic in the face and evaluation of ambiguous and imprecise situations provides a possibility that includes the power of creativity and human understanding of ambiguous concepts. Fuzzy logic can be One of the key elements in risk assessment^17,18^. In Clemen et al.^19^and Mirza et al.^20^used fuzzy logic to estimate the probability of failure in the error tree. Therefore, in order to overcome the limitations of the conventional Bow-tie approach, uncertainties must be minimized. One of the important approaches in minimizing uncertainties is to use the experts’ opinions in combination with the fuzzy set theory approach to estimate the probability of occurrence of basic events. Various studies have examined this issue, such as Jianxing et al.^21^. In another research Srivastava et al. In an article titled Risk Reduction at CNG station using integrated Fuzzy technique to identify and prioritize risks at a gas station using a fuzzy model-based inference model for FMEA Risk Ranking. The results of this study show that the fuzzy approach effectively discovers potential failure and helps manage risk and maintenance decisions^22^. Given that Bow-tie is not a complete way to quantify the risk, updating old data and modeling complex relationships between safety barriers and accidents. Also, it cannot update past probabilities based on new information and data^23^. Therefore, the use of methods that can reduce these defects is one of these methods is the method of Bayesian networks.

### Bayesian network

Although the mentioned methods reduce uncertainty, their structure is static, and there is no possibility of deductive reasoning. In recent years, efforts have been made to address these two problems. Ferdous et al.^24^ use new and dynamic methods such as the Bayesian networks Method, Opinion of Evidence, Monte Carlo Models, and Marco Method as part of these efforts. Among these methods, the Bayesian Networks method is superior to other methods because of its unique features in risk assessment and solution and incident investigation^25,26^. Bayesian networks are classified algorithms based on machine learning that can be used for causal reasoning and risk forecast analysis^27^. Bayesian networks offer several advantages over conventional regression-based methods. Bayesian networks are a graphic representation that includes DAG and conditional probability tables (CPT)^28,29^. Studies related to the Bayesian network include Eskandari et al.^30^, in a study of fire and explosion analysis in industrial processes using fuzzy and Bayesian Networking model optimized and modeling audio absorption in Yucca composites based on compressor gas leak analysis. Using the Bow-tie, 24 primary events, and 11 intermediate events, the results showed that based on the Bayesian network analysis and fuzzy network defects in filtering are the most critical factors in the occurrence of compressor gas^30^. In this research, for a more detailed and reliable explosion risk, a grid-based risk mapping method based on the Bayesian network was provided. Furthermore, three kinds of data-practical information, computational simulations, and subjective judgments were involved generally, and a method was proposed to deal with complex conditions^31^.

### Consequence modeling

In modern safety approaches, consequence prediction or modeling is an essential part of the disaster prevention program to reduce potential losses and determine the severity of the consequences. Consequence modeling is mainly done using mathematical equations. Studies have recommended consequence modeling as an appropriate tool for designing the safety of hazardous industrial units^32,33^. Consequence modeling refers to the quantitative evaluation of the results or potential impact of a particular event or scenario. Consequence modeling is crucial in various fields, including risk assessment, environmental impact assessment, and decision-making processes. Consequence modeling allows stakeholders to make informed decisions and prioritize safety measures based on anticipated results^34^. Consequence modeling is critical in retrofitting buildings from explosions or fire protection for various hazardous industries, including oil and gas, petrochemicals, and energy transfer^35^. A review of literature shows that most research studies have focused on CNG tanks of vehicles, including the study of Rastimehr and others have paid attention to the consequences of methane gas expansion in a fuel station and its situation in its surroundings. This study was conducted in Isfahan province based on ALOHA software and six scenarios. The results indicated that Jet Fire was the most dangerous scenario, which involved not only the CNG station but also the municipal parking area^36^. The risk assessment results indicate unacceptable risk in most affected areas. Additionally, the Bayesian network and PHAST modeling can be useful in the urban design phase and preventing the occurrence of events in fuel stations^37^. The study by Ma and Huang on petrol stations in 2019 showed that petrol stations close to residential areas can cause loss of people. It was conducted by the Bayesian network based QRA method to model initial release to consequent explosions. The results demonstrated that the scenario of release can lead to the loss of humans, which can be influenced dramatically by ignition sources^38^. In 2010, Luo Yong and Guo Xiuchun evaluated CNG explosion and inflammation radius of CNG station and assessed the explosion of CNG stations in the event of an explosion, as well as conditions for the evacuation of people. In this study, the main factors that could cause a fire and explosion were identified. According to calculations of the explosion risk, the safe radius for residential areas is 20.1 m^3^.

**Figure 1:**
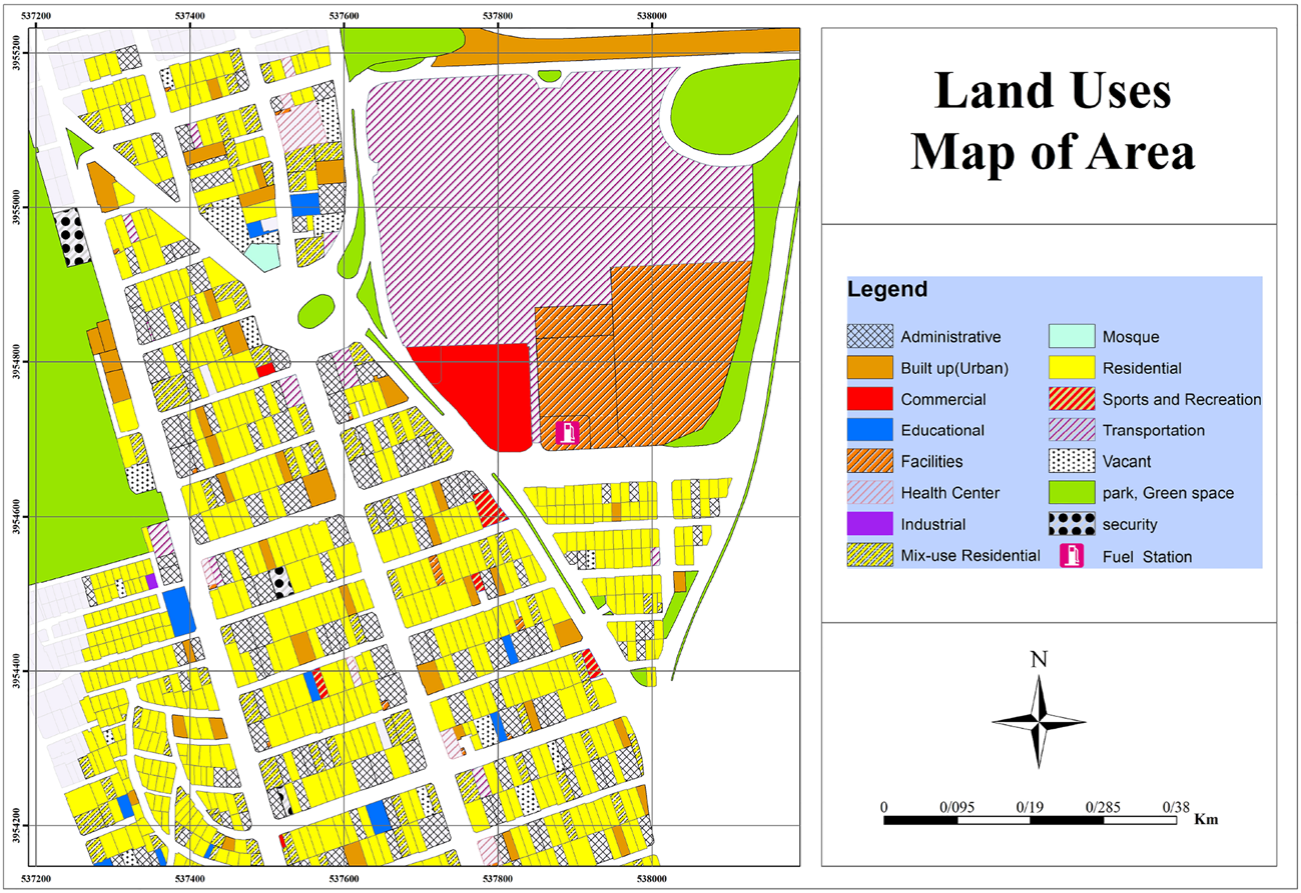
Land use map around fuel station.

This study aimed to provide a method for quantitative risk estimation of CNG stations using a combination of Bow-tie diagrams, fuzzy Bayesian networks, and consequence modeling in a fuel station. Firstly, in order to identify the basic events and build a Bow-tie diagram, a team consisting of safety and process experts was formed. Then, Due to the lack of a proper baseline for determining the fault rate of base events in fault trees in gas stations in Iran, this study used the fuzzy theory method. According to the removal of Bow-tie tree defects, Bayesian networks were used to estimate the probability of final consequences and sensitivity analysis. Finally, according to the values obtained from the fuzzy Bayesian network and fault tree, the frequency of the main event entered the safety software, and the individual and collective risk in the affected area was estimated. This step aimed to determine the risk in the current state and the amount of individual and collective risk in case the most critical underlying events identified were eliminated. This study was conducted in a fuel station. The fuel station is a multipurpose land use, which is next to an LPG (liquid petroleum gas) fuel site. The site is in an area of about 3000 m^2^. The fuel station is located in the vicinity of various land uses, including Shahrvand Chain Store, a recycling center, the waste management center, residential areas, the power station, the terminal park (Fig. 1). The PHAST software (ver. 8.2) was used for modeling explosion. According to the results, the highest vulnerability was observed in CNG station and waste disposal center, and the lowest in residential and administrative land uses.

## Materials and methods

**Figure 2:**
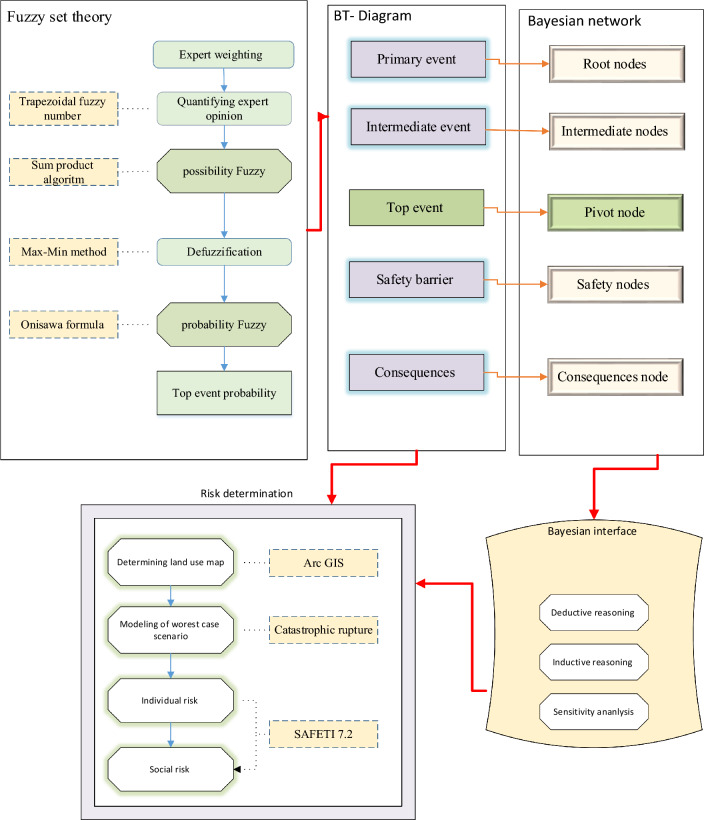
Methodology of research.

Considering that natural gas is mainly composed of methane, to calculate the explosion hazard of CNG stations in this study, methane gas was considered as a flammable substance^39,40^. Figure 2 shows the methodology of the research.

### Bow-tie diagram

The Bow-tie diagram includes primary events, secondary events, and iterations showing the causes and consequences of the main event. This helps visualize the causal relationship between events leading up to a major event and provides a thorough understanding of the risk landscape. This diagram includes a central event that represents a superior event or danger and two branches extend. The left branch indicates reasons or threats that can lead to a superior event, while the right branch represents the consequences or effects that a dangerous event can cause Each branch of the chart is further divided into different layers and represents various contributing factors and risk-related control measures. This model allows comprehensive risk analysis and helps identify preventive and mitigating measures. The Bow-tie diagram provides a clear and structured representation of the risk scenario and makes it easier for stakeholders to understand and communicate the risks involved^11,41^.

### FST

Given that in this study no information was available for the identified basic events, the occurrence probability of the initial event was estimated using the experts’ opinions and fuzzy theory. This study utilized the experts’ opinions and inferences and FST to determine the probability of basic events. According to Mirza et al.^20^, when there is not enough information, experts’ opinions can be used to determine the probability. Three general indicators were considered for selecting the experts including the amount of articles published by the individual, the experience of conducting similar studies, and verifying the expert’s qualification by others^42^. Based on the criteria of Table 1, the relative weight score of each expert was calculated by adding the total obtained points divided by the total points obtained by all experts.

**Table 1:** Criteria for determining the weight score of the experts.

Row	Title	Score	Experience (Year)	Score	Education level	Score	Age (Year)	Score
1	Professor	5	≥ 30	5	Ph. D	5	≥ 50	4
2	Junior academic	4	20–29	4	Master’s degree	4	40–49	3
3	Engineer	3	10–19	3	Bachelor’s degree	3	30–39	2
4	Technician	2	6–10	2	Diploma	2	≤ 530	1
5	Worker	1	≤ 5	1	High school	1		

#### Determining the weight of experts and quantifying their opinions

To quantify the experts’ opinions and determine the weight of their opinions on the failure rate of basic events, based on the study of Saaty and Ozdemir^43^, seven items including "very low, low, relatively low, medium, relatively high, high, and very high" were used. There are various fuzzy membership functions to fuzzify items, including triangular, trapezoidal, bell-shaped, and Gaussian. Considering that in the evaluation and risk estimation studies, zoning and triangular methods are commonly used, such as the study of Mardani et al.^44^ Yazdi et al.^45,46^, which have used triangular and trapezoid fuzzy numbers. In this study, based on the study of Mirza et al.^20^, trapezoidal fuzzy numbers were used. The experts’ opinions were quantified using the method proposed by Chen et al.^47^.

#### Converting linguistic terms into crisp failure possibility (CFP)

The experts’ opinions in the form of linguistic terms were required to be converted to fuzzy numbers. Then, they were converted to a final number called failure probabilities (FPs). Studies have used various techniques for consensus of experts, such as linear opinion pool, max-min Delphi, sum product, and max-product. In this study, the sum-product algorithm were used for the consensus of the experts^24^.

#### Defuzzification

In this study, the max-min method proposed by Chen et al.^47,48^ was used. The max-min FST has been used in several studies, including Anwesa et al.^49^, Shi et al.^50^. In this study, to diffuse the obtained data, the method provided by Yazdi et al. and according to the Equations 1 was used^45^.

1 $${\text{FPS}}({\text{Z}}_{{\text{i}}} ) = [{\text{FPS}}_{{{\text{Right}}}} ({\text{Z}}_{{\text{i}}} ) + 1 - {\text{FPS}}_{{{\text{Left}}}} ({\text{Z}}_{{\text{i}}} )]/2$$

#### Top event and failure probability

The number obtained from the defuzzification step for each event was CFP. The number obtained from the previous step should be converted from possibility to probability. Equation (2) was used to calculate the failure probability of events occur-once^51^.

2 $$FP = \left\{ {\begin{array}{*{20}c} {\frac{1}{{10^{K} }}} & {{\text{FPS}} \ne {0}} \\ 0 & {{\text{FPS}} = {0}} \\ \end{array} } \right.K = \left[ {\frac{1 - FPS}{{FPS}}} \right]^{\frac{1}{3}} \times 2.301$$

In this equation, FP is the probability rate of each basic event, CFP is the possibility number obtained from the defuzzification step, and K is an intermediate variable that is a function of CFP.

### Bayesian network modeling

The Bayesian network is composed of a series of nodes and lines of communication between them. The nodes represent the modeled variables and the lines represent their relationship. According to Jensen^52^, a Bayesian network is the Directed acyclic graphs of DAGs that are considered as a single system. After drawing a Bow-tie diagram to eliminate defects, the constructed model was quantified. For this purpose, the probability values of initial events from fuzzy theory were considered as Marginal probability values of corresponding root events.

#### Deductive reasoning

The ability of Bayesian network deductive reasoning is vital in dynamic risk analysis. Deductive reasoning allows updating the probability of basic events and reduces uncertainty in the model and the results obtained. This makes the model data built closer to reality. By updating the probability of basic event and final consequence, it is possible to choose the most critical, influential base event with the largest share of the main event^30^.

#### Sensitivity analysis

In this study, the ROV method did sensitivity analysis and identification of the most crucial event. This criterion is used to compare the probabilities of the preceding and posterior base events and to select the most critical of the basic events using the rate of change measurement (Rate of Variation) method calculated using the Eq. (3)^53^.

3 $$ROV(BE_{i} ) = \frac{{\pi (BE_{i} ) - \theta (BE_{i} )}}{{\theta (BE_{i} )}}$$

In this regard $$\pi (BE_{i} )$$, posterior probability of the base event., and the $$\theta (BE_{i} )$$$$BE_{i}$$: $$BE_{i}$$ is the prior probability of the base event.

### Consequences modeling

#### Determination of land use map

In order to prepare land use maps, various measures were taken so that after determining the location of the studied area in terms of safety, strategy, and proximity of incompatible land uses, the initial shapefile was prepared by the municipality of the studied area. The research team used the Google Earth Pro 7.3.6 software to determine the exact location of the study area and collected information about the land uses. Then, in GIS software version 10.4, descriptive and spatial information of each land use was completed to investigate the explosion wave in the land uses around the gas station according to the obtained information and satellite image of some of the surrounding land uses. Using atmospheric parameters, vessel specifications, the location of buildings in the area and the adjacent land uses, CNG station explosions were modelled by the PHAST/SAFETI (V8.2) software. Next, by importing the area map with dxf format in the PHAST model, the explosion wave modeling based on different variables and climate conditions of the region and determining the explosion model was carried out in the next step, the final modeling, the shapefile output was extracted and by importing the blast wave and its overlap on the land uses of the studied area in the GIS software, analysis of the risks and damages caused by the explosion wave on the land uses of the study area were investigated. In the next step, based on the standards vulnerability level in Table 9, the amount of deterioration caused by the blast wave was analyzed. In the final stage, the study area map and the overlap of the blast wave were prepared based on 300 dpi.

#### Scenario modeling

After considering all the factors affecting the scenario, it was simulated at this stage, and then, the consequences of incident scenarios were predicted using mathematical models and considering practical factors. After selecting the logical scenarios, the most critical factor in performing the correct analysis of the consequence is to choose the correct model that can simulate the accident as close to the actual conditions. Different studies have introduced valid models for modeling, release, explosion, and flammability^54^. In this stage, considering the number of vessels and the volume of each vessel, the total gas storage capacity of the station was calculated. Given that there are 27 vessels at CNG station, and each vessel has a capacity of 125 liters, the total capacity of vessels at the station is 3.375 liters, Compressor capacity (Nm^3^/h), vessel temperature and pressure was 15(°C), 250 bar. For the BLEVE scenario, a standalone vessel was first defined next to the CNG vessel. Considering that LPG contains 90% propane and 2.5% butane, an LPG material was created in the model by combining propane and butane^55^. Then, the characteristics of the LPG vessel of the model were introduced, including the vessel volume, temperature, pressure, and the type of flammable material. Then, the physical properties of the vessel were entered in the model, including the vessel geometry, radius, and height and vessel volume. Table 2 shows the LPG vessel data in the model.

**Table 2:** Specifications of BLEVE explosion scenario at CNG station.

Temperature (°C)	Pressure (atm)	Volume (m^3^)	Vessel shape	Vessel height(m)	Vessel radius (m)	Explosion type
25	10	200	Spherical	1	4	BLEVE

To determine atmospheric parameters of the study area, it was assumed that the environment is stable. The average temperature and wind velocity over the past 11 years (2007–2017) were calculated. The relative humidity of the area was estimated at 50%. The average monthly temperature and wind velocity over the past 11 years were 18.44 °C, and 3.01 m/s, respectively.

### Risk determination of explosion

#### Social and individual risk

Individual risk means the probability of a person being injured near the accident site. It depends on various factors such as the type of injury, the probability of the accident, and the intense of the accident. In order to calculate the individual risk around the accident site, it was assumed that all the final consequences in accidents or events are cumulative^16^. Social risk is a measure of the population’s risk that is close to the hazard. F-N curves are used to present social risk and the sum of the repeatability of the consequences caused by the accident is drawn in logarithmic form in terms of the number of casualties caused by the accident^16^.

## Results

### Bow-tie diagram of CNG leakage

**Figure 3:**
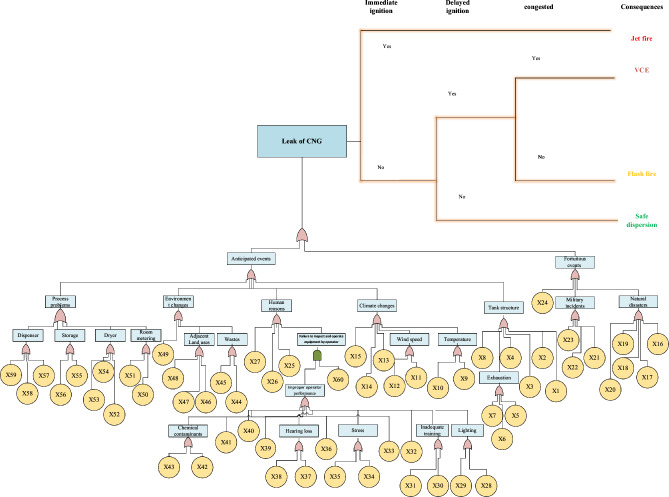
Modeling of the CNG explosion scenario using Bow-tie diagram.

Based on the investigations carried out at the fuel station site, this study prepared intermediate events and the basic event according to the occurrence of the CNG tank leakage. Bow-tie diagram was drawn which is shown in Figure 3.

In this research, the top event was the leak of CNG. To determine the probability of the top event, the probability of basic events was calculated, then, to determine the probability of basic events, a seven-scale approach was used. First, to determine the probability of the occurrence of basic events, seven qualified experts were selected, including the chief operations inspector, HSE expert, facilities expert, HSE manager, process expert, and two faculty members. The relative weight of the selected people was calculated according to the previously mentioned method. The specifications of the selected experts and the weight scores of each are presented in the Table 3.

**Table 3:** Weighted scores of the experts.

Experts	Title	Experience	Education	Age	Expert weight
1	5	5	4	4	0/234
2	4	4	4	3	0/078
3	3	3	3	2	0/156
4	2	2	2	1	0/187
5	1	1	1	3	0/187
6	5	2	5	2	0/093
7	5	3	5	2	0/062

### Fuzzy set theory

The experts’ opinions were evaluated using a questionnaire and a seven-scale approach. From these findings and interviews with experts, the probability of 60 basic events (X) and 25 intermediate events was determined in the form of designed forms. Table 4 reveals the basic events with the symbol X and the description of each of the basic events and the degree of probability obtained from the fuzzy theory are presented.

**Table 4:** Description of basic events and probability obtained from fuzzy.

Token	Basic event	Probability of failure	Token	Basic event	Probability of failure
X1	Not up-to-date technology	0.00175388	X31	Requirements for conducting training classes by managers	0.00133352
X2	Lack of maintenance	0.00166341	X32	Fatigue	0.00102329
X3	Unsafe equipment	0.00174181	X33	Shift work	0.0012942
X4	Type of ignition material	0.00151356	X34	Stress (internal causes)	0.00151356
X5	The nature of the chemical substance	0.00149968	X35	Stress (external causes)	0.0012942
X6	Inspection defect in wear detection	0.00149968	X36	Not having enough experience and skills	0.0015417
X7	Improper use of the equipment	0.00149968	X37	Hearing loss (non-occupational causes)	0.0012331
X8	Leakage	0.00141579	X38	Hearing loss (occupational causes)	0.00120781
X9	High temperature	0.00125603	X39	Failure to notify the control room in time	0.00185353
X10	Low temperature	0.00110154	X40	Fear of explosion and fire by operator	0.0013213
X11	Horizontal wind speed	0.00157036	X41	Operator performance (temperature and humidity)	0.0012331
X12	Vertical wind speed	0.00133352	X42	Chemical pollutants (particles)	0.00103276
X13	Environmental stability and instability	0.00148594	X43	Chemical pollutants (gas and steam)	0.0010666
X14	Sunny hours	0.0011722	X44	Solid waste	0.00115878
X15	Relative humidity and evaporation rate	0.00140281	X45	Liquid waste	0.00108893
X16	Lighting	0.0013213	X46	Adjacent commercial use	0.00130918
X17	Landslide	0.0012331	X47	Adjacent residential use	0.00115878
X18	Flood	0.00128233	X48	Adjacent industrial use	0.0012942
X19	Earthquake	0.00148594	X49	Land uses changes	0.00142889
X20	Land settlement	0.0012942	X50	Room metering (measurement of changes)	0.00127057
X21	Deliberate vandalism	0.00145546	X51	Room metering (operator error	0.0011246
X22	Incidents related to the missile site	0.00142889	X52	Lack of standard dryer quality	0.0013213
X23	Military attack	0.00155597	X53	Disturbance in the electricity flow of the dryer	0.00107895
X24	Explosion of other equipment	0.00115878	X54	Fire dryer heaters	0.0011246
X25	Deliberate error in the execution of the recipe	0.0012331	X55	Leakage of tank	0.0012942
X26	Accidental collision valves	0.00083946	X56	Adjacent tanks	0.00108893
X27	Failure to issue a work permit	0.00164816	X57	Dispenser leakage and damage	0.00130918
X28	Artificial lighting	0.00138676	X58	Disregarding dispenser safety signs	0.0010666
X29	Natural lighting	0.00144212	X59	Dispenser malfunction	0.0013213
X30	Lack of cost	0.00151356	X60	Improper management performance	0.00016326
Immediate ignition	0.00149968	Delayed ignition	0.00211349		
Congested	0.0011722	Top event: leaked of CNG	0.054831905		

In the next stage, the repetition of the intermediate events was estimated in order to determine the probability of basic events using fuzzy theory. Table 5 shows the levels of unexpected and non-extended events. The intermediate events of the tank structure had the highest probability of failure, and natural disasters had the lowest probability.

**Table 5:** Probability of failure of intermediate events in expected and unexpected events.

The probability of failure	The intermediate events		Row
0.009566	Process causes	Expected events	1
0.007415	Environmental causes		2
0.0052	Human causes		3
0.009285	Climate changes		4
0.012491	Tank structure		5
0.006599	Military threats	Unexpected events	6
0.004433	Natural disasters		7

### Bayesian network modeling

**Figure 4:**
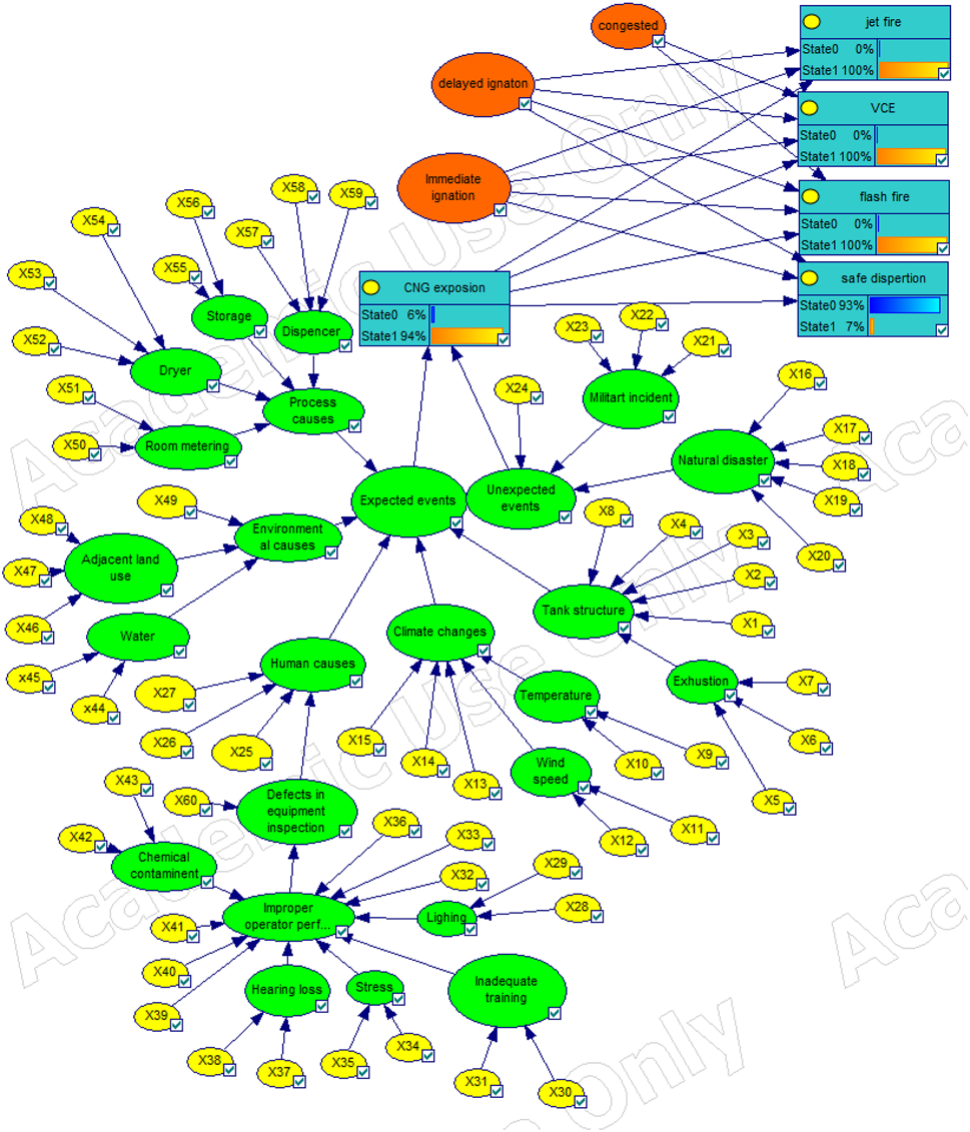
Modeling Bow-tie diagram on Bayesian networks.

According to Figure 3 presented algorithm, the Bayesian network for CNG gas leakage and its consequences were plotted according to Figure 4. Genie software was used for this purpose. The probability of the main event, gas leak, was estimated at 5.57157e−2. This amount of Bow-tie method is 0.054831905. The probable results of the final consequences are shown in Table 7 using the Bayesian network model. Based on these results, the most likely chance of the Flash Fire's consequence is 1.96983E−3. Table 6 shows the conditional probabilities of the occurrence of the final consequence. Table 6 shows how to quantify the consequences based on Immediate ignition, delayed ignition, and congestion possibilities. For example, jet fire occurs in the case of gas leakage without a delayed ignition source and an immediate ignition source. Flash fire occurs in the uncondensed environment in case of gas leakage, a delayed spark source, and the absence of an immediate ignition source.

**Table 6:** Probability of conditional final consequences probability.

Consequences	Immediate ignition	Delay ignition	Congested
Jet fire	1	0	–
VCE	0	1	1
Flash fire	0	1	0
Safe dispersion	0	0	–

#### Inductive reasoning

The ability to select the most influential primary event that contributes most to Eli's event can be identified by updating the possibility of root events and final consequences. Table 7 shows the probable values of the up-to-date consequences on the Bayesian networks. As can be seen, the consequence of a Flash fire with 1.96983E–3 has the highest final consequences.

**Table 7:** The probability of final consequences on Bayesian networks.

Consequences	Bow-tie	Prior probability	Posterior probability
Jet fire	8.22303E−5	1.5600408E−6	2.8E−5
VCE	1.35639E−7	1.2085859E−7	2.1692E−6
Flash fire	1.15577E−4	1.097505E−4	1.96983E−3
Safe dispersion	5.4633962E−2	7.0797849E−2	0

#### Sensitivity analysis

**Figure 5:**
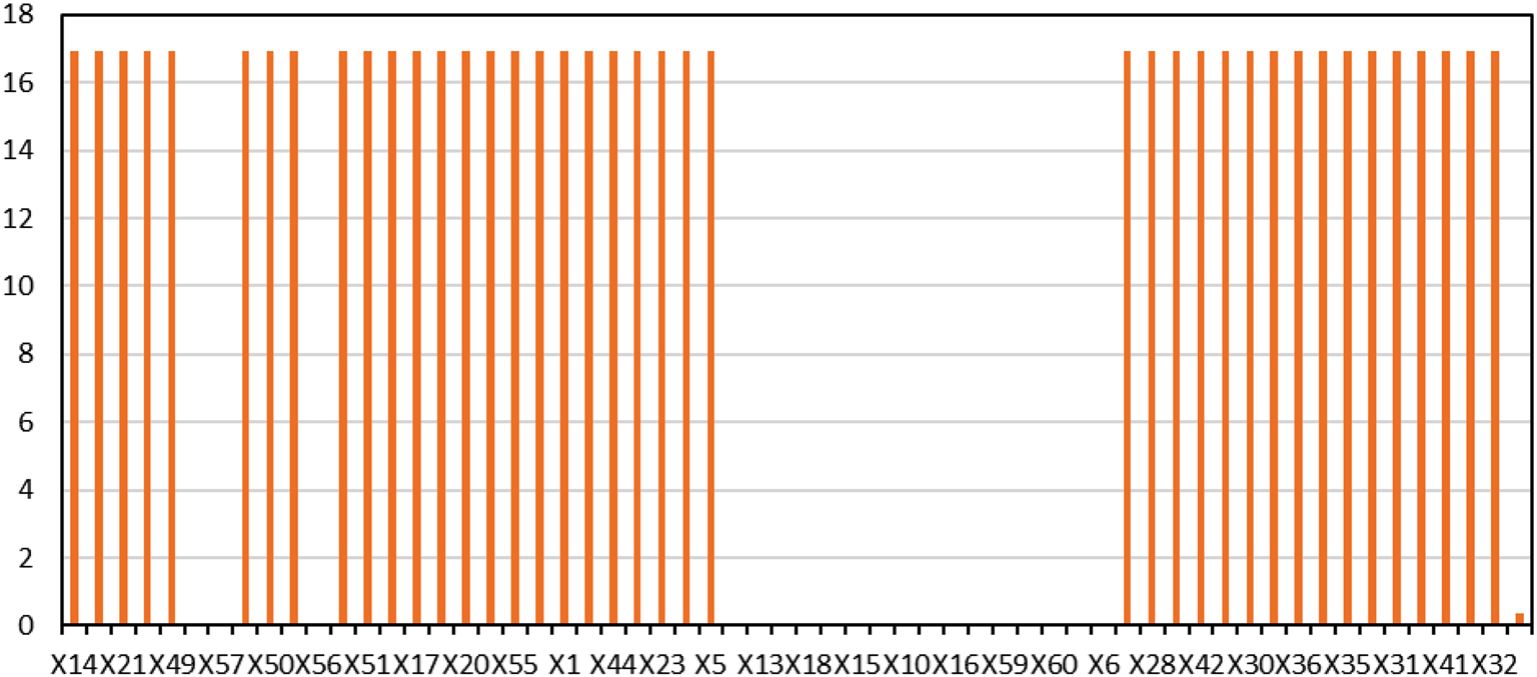
Sensitivity analysis of basic events in Bayesian networks.

This study used the ROV method to analyze the sensitivity of Bayesian networks and identify the most critical underlying event. Figure 5 shows the results of basic event updates using ROV criteria. Following the events were identified as the most critical root event in the occurrence of the main scenario. Then, the X14 event and the X8 and X21 are in the next rank, respectively. After identifying the most critical events, their effect on the probability of the main event was investigated. For this purpose, the X14, X8, and X21 events were considered based on Table 8. Accordingly, the time of all three critical events identified as zero was considered. The amount of the main event, namely CNG gas leakage, was estimated at 5.188723E−2.

**Table 8:** Critical event control results on final consequence and main event.

Bayesian Network Approach (Probability)				Control measure
Leak of CNG	Jet fire	VCE	Flash fire	
5.57157e−2	1.56004e−6	1.20858e−7	1.097510e−4	No measure
5.546075e−2	1.52901e−6	1.184547e−7	1.075676e−4	X14 control
5.543769e−2	1.52255e−6	1.179541e−7	1.071133e−4	X8 control
5.433937e−2	1.521502e−6	1.178729e−7	1.070393e−2	X21 control
5.188723e−2	1.452842e−6	1.125537e−7	1.022090e−2	X14, X8, X21 control

### Consequence modeling

**Figure 6:**
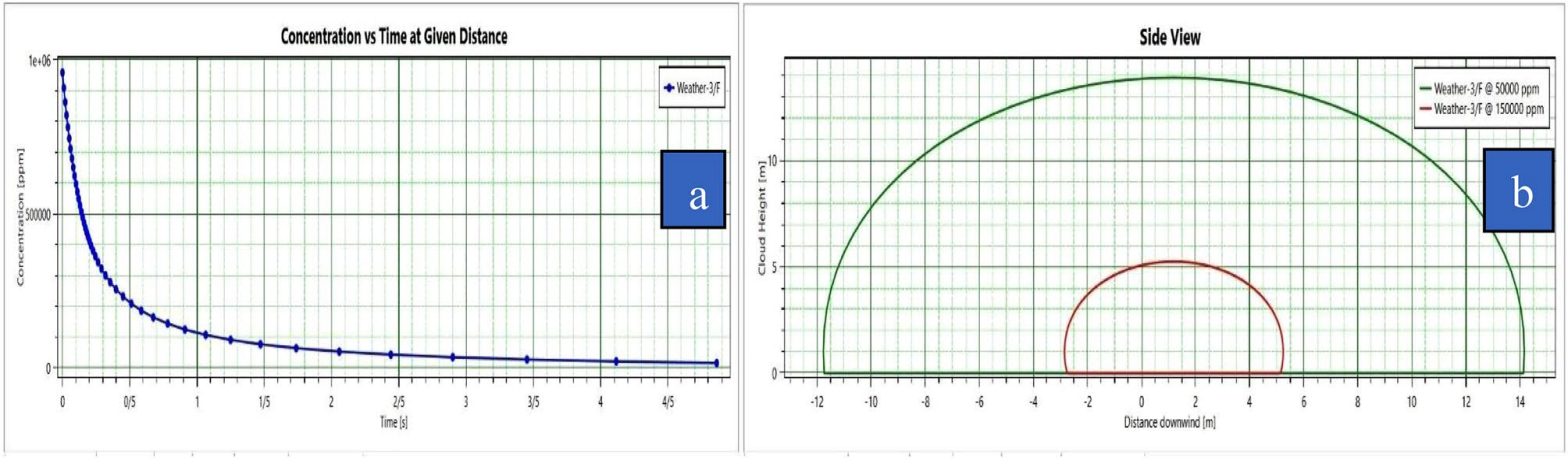
(**a**) Concentration vs. time at a given distance (**b**) side view of the vapor cloud.

Figure 6a represents that the cloud vapor concentration was about 960,000 ppm in the first few seconds and its concentration began to drop to 10,000 ppm. The vapor cloud concentration varied over time. The modeling results show that vapor cloud dispersion took more than 4 s. Figure 6b reveals the side view of the vapor cloud movement in the area. The vertical axis represents the vapor cloud height at concentrations of 50,000 ppm and 150,000 ppm. The horizontal axis represents the vapor cloud distance from the source in the wind direction

### Effects of accident scenarios assessment

**Figure 7:**
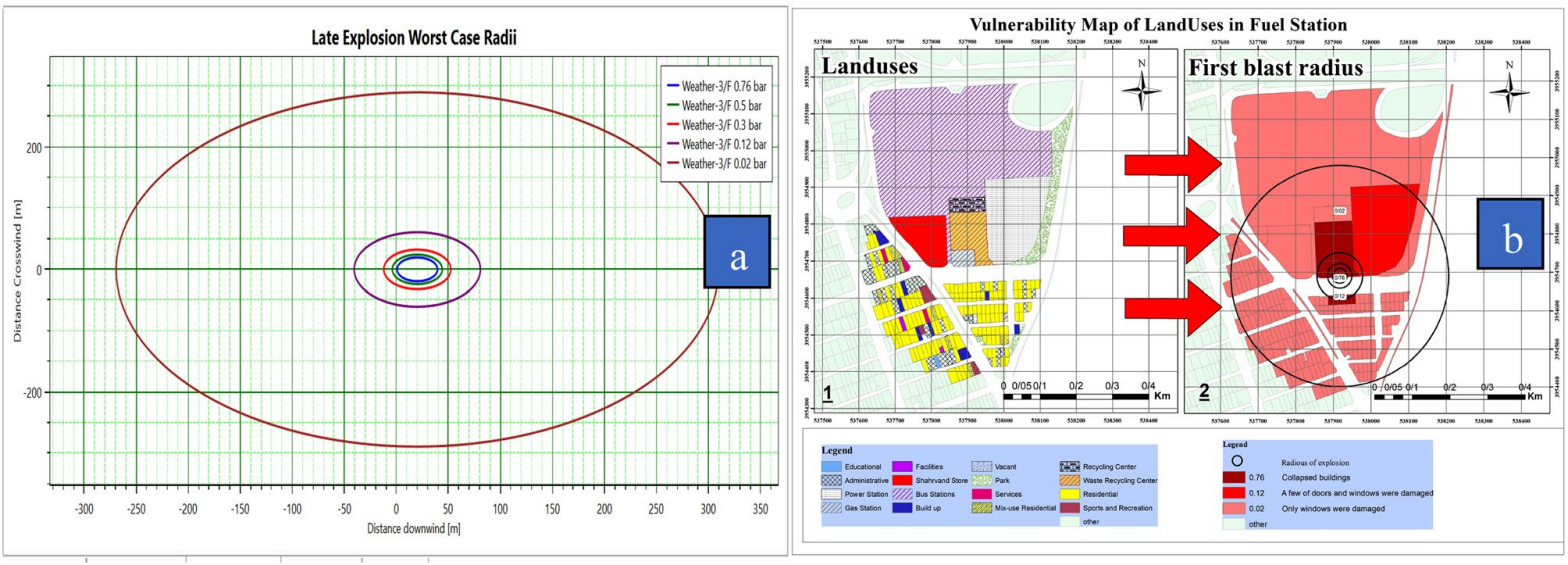
(**a**) Diagram Late explosion, (**b**) Vulnerability map of land uses.

Figure 7a shows the radius of vapor cloud explosion at various distances. The blast wave at different pressures was associated with different power degradations. According to the blast wave pressure at different distances, and vulnerability standards^56^, a comparison was made between the blast wave-induced damages in the surrounding buildings. Figure 7b shows that the greatest effects of vulnerability of land use in the blast wave radius can be observed at pressures of 0.76, 0.12, and 0.02 bar. For this reason, the overlap between the blast wave radiuses was investigated at these pressures.

Concerning Figure 7b, by comparing the location of land uses and the blast wave radius of the cloud gas explosion, the vulnerability of neighboring land uses to the explosion at different pressures was illustrated. According to the first blast radius, the highest and lowest levels of vulnerability in adjacent land uses were observed at pressures of 0.76 and 0.02 bars, respectively. Table 9 shows the vulnerability level of land uses affected by the blast wave.

**Table 9:** Vulnerability of land uses adjacent to CNG stations to the first explosion.

Land uses	Number of land uses	Pressure	Vulnerability
Gas station, waste management center	2	0.76	Complete destruction of buildings
Gas station, waste management center, power station, administrative, residential	9	0.12	Door and window damages
Administrative and educational centers, facilities, commercial buildings, vacant lots, built up, park, services, mix-use residential, residential, sport, gas station, waste management center, power station, recycling center	209	0.02	Window damages

**Figure 8:**
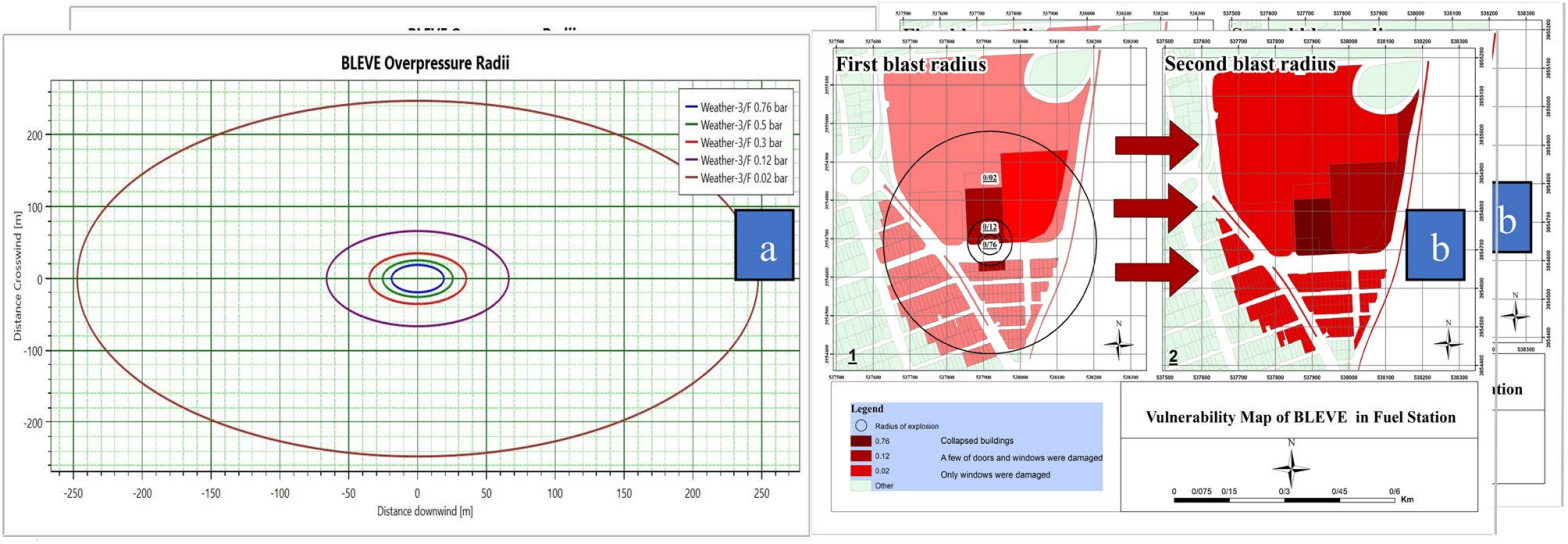
(**a**) Blast wave radius (**b**) Vulnerability map of BLEVE overpressure.

Figure 8a shows the value of the blast wave at different distances, and the highest pressure at a distance of 20 m was equal to 0.75 bar and the lowest pressure at a distance of 247 m was 0.02 bar. Figure 8b shows the effects of vapor cloud blast wave and BLEVE explosion. Although the pressure of the blast wave is the same in both forms, according to the radius range of the second blast wave, in this case, it had a high level of vulnerability.

The vulnerability level of land uses surrounding the fuel station in terms of distance and pressure. By increasing the distance from source of explosion, the level of damage to land uses decreased. At a distance of 39 m from the first explosion and 20 m from the second explosion, the greatest damage occurred in the CNG station and the recycling center. Also, the lowest level of vulnerability was observed at a distance of 310 m from the first explosion and 247 m from the second explosion.

### Risk determination

Based on the strategic location of the area and the high density of residential buildings and the different land uses, the information related to the model are shown in Table 10. The HSE UK is used to rank individual risks because this standard is a comprehensive standard for assessing and managing health and safety risks in the workplace; this standard fully addresses various risk factors such as risk identification, risk assessment, risk control, and monitoring. As a community standard of articles and studies in the field of risk assessment, this standard is used as a reference for risk assessment^57^.

**Table 10:** Data about analysis and reaching the risk.

Scenario	Stability of class	Density	Population	Fraction of population indoors for societal	Ignition probability (fraction)	In time period (s)
Catastrophic rupture	3/F	0.00180126	200	0/9	1	10

**Figure 9:**
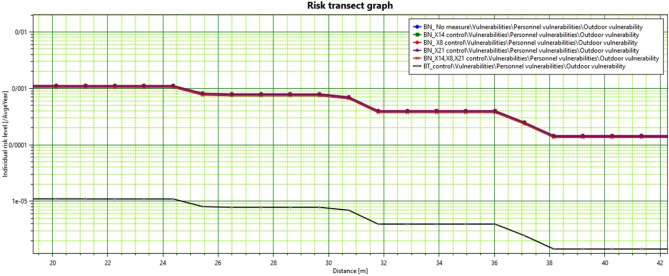
Individual risk contours caused by the CNG Leakage.

In the present study, the Safety 8/2 software was used to calculate individual risk and social risk contours. According to the scope of this study, risk assessment was conducted with the objectives and goals of the study and the risk criteria. This purpose, the estimated repetition was used in Bayesian network and Bow-tie diagram. First, the amount of recurrence calculated in the Bayesian networks was used without any control at basic events to estimate risk. Then, the events were controlled due to the sensitivity analysis and critical events identified. Assuming zero of these critical risks separately and together, the estimated repetition value was used to estimate the risk in SَAFETI software. Figure 9 shows individual risk in various modes, including controlling basic events and the X14, X8, and X21 events on the Bayesian and the Bow-tie diagram networks. As can be seen, in the area calculated in the Bow-tie method for estimating the risk of the individual risk, the individual risk value is less than the mode in the Bayesian networks. The risk estimated in the Bow-tie method is up to 24 m from the site of 10E−5, and for Bayesian networks, this value was 0.001.

**Figure 10:**
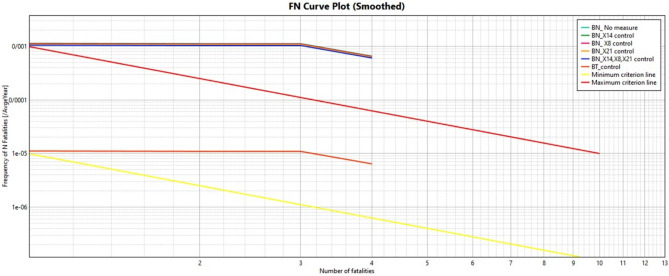
The social risk caused by the CNG Leakage.

Figure 10 illustrates the overall risk associated with the explosion of the CNG tank. The presence of residential areas near the gas station, a high population density, a significant amount of flammable materials stored in the tanks, improper operating conditions, and incompatible land uses can lead to severe damage and accidents. The estimated collective risk was in the unacceptable range, amounting to 10e−3. Figure 10 shows the collective risk estimated in the Catastrophic scenario of the CNG tank based on modeling the consequence and the repetitive results of the Bayesian and Bow-tie networks. As it is, the collective risk estimated is based on the repetition calculated by the Bow-tie 10E−5 while estimated in Bayesian networks for all three modes of 0.001.

## Discussion

The study used the Bow-tie technique to analyze the quality and quantitative cause of the risk and the probability of risk. Figure 3 shows the display of this method. The causes of events were examined in two categories including expected and unexpected causes. In the Bow-tie, a total of 85 causes or defects in the CNG tank explosion were introduced as the top event of the Bow-tie, and 60 basic events and 25 intermediate events were introduced. Four final consequences were identified depending on the type of substance involved in the incident: Jet fire, Flash fire, VCE, and safe release. The results showed that the leak of CNG gas was 0.054831905 a year. The probability of process, environmental and storage structure at the top event were 0.009566, 0.009285, and 0.012419, respectively, which are the three high probability events in the top event, and have the most reservoir structure in the tank explosion. In other words, the leak of CNG gas may occur once every 80.5 years due to a defect in the reservoir structure. There have been numerous studies in this field. In the study of Rajakarunakaran et al.^58^ an LPG gas was examined based on the 16 basic and four intermediate events in the study of Ouache Rachid et al.^59^ 6 basic and 2 intermediate events were identified. Type of tank, fluid containing it, presence of different accessories in each of the LPG tanks Reservoir volume، expansion of base events, consideration of different physical boundaries in a variety of studies، number of unauthorized occurrences and concentration level can be the major difference in the number of base- and mid-overflow events detected. Classic Bayesian networks always use definitive numbers to possibly basic events. Finding definitive numbers for basic events, especially in the CNG tank, is virtually impossible due to the lack of a database. In this regard, Ren et al.^60^ stated that three types of uncertainty (including ambiguity, random, and lack of knowledge) can be reduced by using fuzzy Bayesian networks. However, using definitive numbers for root events and safety barriers leads to uncertainty in the structure of fuzzy Bayesian networks. Although the fuzzy theory can reduce uncertainty, it cannot deserve analog reasoning in the structure of fuzzy methods such as the fuzzy fault tree method, fuzzy Bow-tie model, and consequence models^61^. The combination of fuzzy theory and Bayesian networks uses fuzzy numbers instead of definitive numbers in the possibility of basic events and reduced uncertainty caused by knowledge, but also one of the unique features of Bayesian networks that use deductive and inductive reasoning.

The results of deductive reasoning of fuzzy Bayesian Networks with the update of Bayesian Networks in the CNG gas leak scenario indicate a sudden fire of 1.96983E−3, identified as the most likely consequence of CNG gas leakage. In both cases, the inductive and deductive reasoning of the jet fire after the CNG gas leak is the most likely consequence. One of the common features of the two the Bow-tie model and the Bayesian network is inductive reasoning. The results of the inductive reasoning of the Bow-tie model showed that the probability of the main event, namely CNG gas leakage, was 5.4831905E−2. However, this value in the Bayesian network is 5.57157E−2, more significant than the value obtained in the Bow-tie model. This dispute can be due to the conditional dependence between events and common causes Identifying critical events that have the largest share in the main event is one of the most essential issues for preventive measures. Identifying critical events that contribute the most to the main event is one of the crucial issues for providing preventive measures. In Bayesian Networks, the increased values of updated probabilities are used to identify the most critical event. This method may provide inaccurate information to risk analysts, leading to misrepresenting the wrong control and preventive measures for controlling the main event. In this case, the results of dynamic risk analysis studies may not be effective.

This study used the ratio of change rate (ROV) probability instead of the probability of up-to-date probability on Bayesian networks. This method showed that the basic events of the X14, X8, and X21 had the highest rate change rates, so that these events will have the most role in the main event (CNG gas leak). Therefore, preventive and targeted measures can be presented.

In this study, PHAST 8.2 software was used to model the consequence. This software is one of the most widely used and reliable software for material leakage modeling^62^. In this study, the essential feature of the PHAST software was the ability to define a mixture of materials, which is a great advantage, especially in the oil and gas industries^63^. The effects evaluated in the present study for the CNG tank include flash fire, jet fire, and vapor cloud explosion. Figure 6a shows vapor cloud concentration versus distance and time of the fuel station. The vapor cloud concentration upon the initial release of flammable substances was at its peak, and its concentration dropped over time. Figure 6b reveals that the vapor cloud was 5 m high at a concentration of 150,000 ppm and 14 m high at a concentration of 50,000 ppm. The vapor cloud concentration was about 150,000 ppm at a distance of less than 5 m and it dropped to 50,000 ppm at a distance of 14 m. The vapor cloud expanded up to a distance of 14 m in the surrounding area. Given that methane gas ignited at concentrations of 50,000–150,000 ppm, there was the probability of inflammation at a distance of 4–14 m from the source. At a distance of 39 m, the blast wave pressure was estimated at 0.76 bar (Figure 7). According to vulnerability level standards of buildings at different blast wave pressure^56^, all buildings are expected to be destroyed at the above distance. At a distance of 39–44 m from the explosion source, the blast wave pressure was estimated at 0.5 bar. According to the vulnerability standards of the buildings, all doors and windows of buildings surrounding the gas station can be damaged. At a distance of 44–52 m, the blast wave pressure was estimated at 0.3 bar. At this distance, most of the doors and windows in the neighboring buildings can be damaged. Meanwhile, up to a distance of 300 m, the land uses around the fuel CNG station were at the risk of damages that may be induced by broken glasses of buildings. However, there was no danger beyond this distance and this was considered as the safety range. Based on Table 9, it can be observed that different cabarets are located at different distances from the blast wave, so it is observed that the most damage b of the blast wave is composed of the gas station and waste center in such a way that the blast wave pressure is at its highest and causes destruction of buildings and inflicts many damages and can also be observed that Moving away from the blast wave, what land uses fall within what range of the wave and what damage they contain. In the studied area and according to Figure 7b, a total of 209 uses can be affected by the last waves, which mostly included residential and office uses. There were 132 residential uses and 95 office uses within the blast wave radius. According to Figure 8a,b, at a distance of 20 m from the explosion source, the pressure was 0.76 bar. This amount of wave pressure can lead to the destruction of all buildings. At a distance of 25 m from the source of the explosion, the pressure was 0.5 bar, damaging all the doors and windows of the buildings around the fuel station according to the vulnerability standards^56^. At a distance of 35 m, the pressure of the blast wave reached 0.3 bar, which can damage most of the doors and windows. At a distance of 66 m, the pressure of the blast was 0.12 bar, which may damage a number of doors and windows. In general, the effects of the explosion may extend up to a distance of 247 m. According to the standard values^56^ at this distance, the pressure of the blast wave was equal to 0.02 bar, which can break the windows of the buildings around the fuel station. In the second explosion (Figure 8a), due to the spread of the blast wave, a large number of land uses, including 95 residential users, 32 office users, a park, a bus station, and an educational center were affected by the blast wave.

In the study of Yong and Guo^3^ on traffic evacuation analysis for CNG explosion and burning radius, the safe radius of residential areas in the event of an explosion was estimated at 20.1 m. However, in this study, the safe radius for residential areas was more than 310 m, which is about 15 times longer than their estimated distance. It is probably due to the number of pressure vessels under climatic conditions and the dual-purpose nature of the station under study that have expanded the radius of blast wave. In the study of Soo-Kim^64^, the radius of blast wave was much more limited, which could be due to the gas leakage. In the study of Badri et al.^65^ on CNG stations in Tehran, the amount of explosion caused by CNG release was calculated by 1 bar. In the above studies, the TNT model was used instead of the TNO model to model the explosion. Due to the differences in parameters and process conditions such as temperature, pressure, leakage size, pipe diameter, and equipment in these studies, with the present study, the difference between the power of the explosion is logical. After calculating the repetition rate of the main scenario and entering the scenarios and population information in PHAST and SAFETI (V8.2) software, individual risk levels were drawn for the explosion scenario and the catastrophe scenario. The results of collective and individual risk estimation (Figs. 10 and 9) based on Bow-tie modeling and Bow-tie modeling are less than 10E−4. In contrast, the determination based on modeling and FBN showed that the risk is estimated to be more than 10E−4. According to a standard, the level of risk should not be more than this amount^66^. Since the estimated risk is above the limit, it is necessary to implement the probability and intensity reduction programs at the studied fuel station.

The results of the Bayesian network show that most deficiencies are due to inadequate supervision and the need for appropriate safety plans at a gas station. Based on these problems, implementing an integrated safety management system at these CNG stations is essential due to its high-risk potential and high population density. According to the standards by the UK Health and Safety Agency, there is a criterion for constructing places with different land uses in the units at risk, such as Iran. Accordingly, the land has been divided into four categories including industrial areas, shopping, and entertainment centers, residential areas, and sensitive organizations and centers. On the other hand, individual risk segmentation ranges are also divided into three zones including the inner area with a 10E−5, the middle-risk area with a risk level of 10E−5 to 10E−6, and the outer area with 10E−6 to 10E−7 risk levels. Safety recommendations would involve avoiding any construction of small industrial units and low-population areas in the indoor area. Also, in an outdoor area, only the construction of sensitive organizations and areas, as well as large, entertainment malls should be prevented^67^. According to the criteria for individual risk acceptance in the European Union and the UK^68^, the results of individual and collective risk assessment in the present study exceed the acceptable amount of neighbors, and this reflects the lack of safe intervals in adjacent locations adjacent to the CNG fuel station. In the present study, the selected station has a strategic and sensitive position in relation to its neighboring land uses. For this reason, unavailability of sufficient data and information to prepare the vulnerability map of the region has been one of the major limitations of this research. Accordingly, future studies can achieve more favorable results by completing data and studying the connections of different land uses with each other.

## Conclusion

Today, the expansion of the human population, on the one hand, leads to the expansion of urban and rural boundaries and, on the other hand, leads to the increasing need for human resources such as oil and gas. This, in turn, has led to the proximity of fuel stations to residential and commercial centers. One of its most important consequences is the location of residential areas within the risk range and increased casualties in the event of an accident. Given the high potential of CNG fuel stations in accidents, and the high intensity of possible accidents, one of the most important ways to reduce human consequences is to predict risk boundaries and determine the maximum progress of residential areas in the vicinity of fuel stations. In this regard, the effects of dominoes caused by the explosion of the CNG reservoir should be investigated.

Risk assessment in fuel stations and determining the effective causes of accidents in urban areas can be an appropriate contribution to risk control. This study used integrated modeling and probabilistic methods to estimate individual and collective risk at the CNG stations. In order to model the probabilistic scenario, PHAST/SAFETY software was used to estimate the probabilities. Also, the Bow-tie n diagram and Bayesian networks were used to draw the consequences. Due to the unavailability of suitable data to estimate the underlying events in the main event, fuzzy theory was employed to mitigate uncertainty and reduce ambiguity. The results of this study showed that the estimated risks of these combined methods are in an unacceptable range. Based on the results of this study, measures can be taken to reduce the effects of possible accidents. Since the studied fuel station is located in an urban area and a high desertification environment, it is necessary to take control measures and develop a response plan for the appropriate emergencies in the agenda of the relevant authorities. The limitations of this study include the lack of accessibility and confidentiality of information about the gas stations, the region's strategic location, and the lack of cooperation of some of the authorities. Based on the results of the study, field inspections, and according to the study area and lack of structural changes in the built sections in the study area, Pre-Incident Planning (PIP) and Emergency response plan (ERP) scenarios can be used to develop a maintenance plan and appropriate training for residents and paying attention to land uses compatibility reduced the risk. Suggestions include paying attention to adjacent land uses in urban areas while constructing compressed natural gas stations to reduce domino accidents and effects, extensive studies, and research on domino accidents in urban areas.

## Data Availability

The datasets used and/or analyzed during the current study are available from the corresponding author upon reasonable request.
